# *S. epidermidis* Rescues Allergic Contact Dermatitis in Sphingosine 1-Phosphate Receptor 2-Deficient Skin

**DOI:** 10.3390/ijms241713190

**Published:** 2023-08-25

**Authors:** Kana Masuda-Kuroki, Shahrzad Alimohammadi, Anna Di Nardo

**Affiliations:** Department of Dermatology, School of Medicine, University of California San Diego, 9500 Gilman Drive, La Jolla, CA 92093, USA; kkuroki@health.ucsd.edu (K.M.-K.); shalimohammadi@health.ucsd.edu (S.A.)

**Keywords:** sphingosine 1-phosphate, sphingosine 1-phosphate receptor 2, *Staphylococcus epidermidis*, 3,4-Dibutoxy-3-cyclobutene-1,2-dione (SADBE), contact dermatitis

## Abstract

Recent studies have identified a subtype of the S1P-receptor family called sphingosine-1-phosphate receptor 2 (S1PR2), which plays a crucial role in maintaining the skin barrier. It has been observed that S1PR2 and *Staphylococcus epidermidis* (*S. epidermidis*) work together to regulate the skin barrier. However, the interaction between these two factors is still unclear. To investigate this, a study was conducted on healthy skin and allergic contact dermatitis (ACD) using 3,4-Dibutoxy-3-cyclobutene-1,2-dione (SADBE) on the ears of *S1pr2^fl/f^*^l^ and *S1pr2^fl/f^*^l^ *K14-Cre* mice and using 1 × 10^6^ CFU of *S. epidermidis* to examine its effects on the skin. The results showed that in *S. epidermidis*-conditioned ACD, the ear thickness of *S1pr2^fl/f^*^l^ *K14-Cre* mice was lower than that of *S1pr2^fl/f^*^l^ mice, and mRNA expressions of *Il-1β* and *Cxcl2* of *S1pr2^fl/f^*^l^ *K14-Cre* mice were lower than that of *S1pr2^fl/f^*^l^ mice in ACD with *S. epidermidis*. Furthermore, the gene expression of *Claudin-1* and *Occludin* in *S1pr2^fl/f^*^l^ *K14-Cre* mice was higher than that of *S1pr2^fl/f^*^l^ mice in ACD with *S. epidermidis*. The study concludes that *S. epidermidis* colonization improves the skin barrier and prevents ACD even when S1P signaling malfunctions.

## 1. Introduction

The epidermis is the body’s outermost layer, directly exposed to environmental stimuli. Therefore, the epidermis provides a barrier function against chemicals, pathogens, and mechanical stress to maintain normal biological processes. Sphingolipids are the essential lipid molecules that form cell membranes and are present in all eukaryotic cells [1]. Sphingolipids and their metabolites play crucial roles in cell signaling, cell survival and differentiation, and immune cell trafficking [2,3]. Sphingosine 1-phosphate (S1P) is one of the sphingolipids derived from cell membranes and acts through G-protein coupled receptors called S1P receptors [4]. The S1P receptor family consists of five different subtypes, S1PR1-5, which reside in various cells and tissue [5], while keratinocytes are shown to express all five receptor types [6,7]. Our previous study found that S1PR2 has a crucial role in maintaining the skin barrier, showing that S1PR2 knockout mice presented a decreased expression of tight junction proteins such as occludin (OCLN) and claudin 1 (CLDN 1), as well as decreased filaggrin (FLG) and zonula occludens-1 (ZO-1), which are key constituents of the stratum corneum barrier [8]. However, it remains unclear whether S1PR2 found in epidermal keratinocytes, which mainly form the skin barrier, or found in cells other than keratinocytes, is responsible for this function.

Meanwhile, it is widely known that, in humans, a large variety of bacteria reside in the skin as commensal bacteria and *Staphylococcus epidermidis* (*S. epidermidis*), specifically, induces skin ceramide production, which protects the skin and contributes to the maintenance of the skin barrier by secreting sphingomyelinase [9]. Therefore, S1PR2 and *S. epidermis* are both involved in controlling skin barrier maintenance. However, the nature of their interaction still needs to be determined.

Allergic contact dermatitis (ACD) is one of the most frequent skin inflammatory diseases caused by repeated allergen exposures. Our data show that the level of S1PR2 in the skin goes up during ACD, which suggests that this increase is a natural response to help repair the skin barrier. An efficient skin barrier is the principal protection against a variety of allergens and can prevent ACD formation [10]. Although a large variety of haptens in chemicals, dyes, drugs, and metals cause ACD, the only treatment option is the administration of corticosteroids and the avoidance of allergen exposures [11]. However, long-term topical corticosteroid treatments increase the risk of adverse effects, including cutaneous atrophy and telangiectasias [12]. This study focuses on the role of epidermal S1PR2 together with *S. epidermidis* in ACD, where the skin barrier is impaired. Our results show that *S. epidermidis* improves the skin barrier and suppresses the inflammation of ACD to compensate for S1PR2 deficiency in the skin.

## 2. Results

### 2.1. S1PR2 Expression Is the Most Upregulated among S1P Receptors in ACD

**Figure 1:**
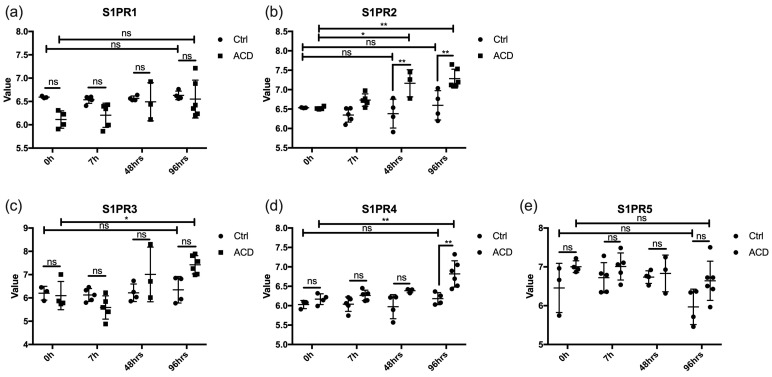
S1PR2 expression increases in contact dermatitis. Gene expressions of S1PR1 (**a**), S1PR2 (**b**), S1PR3 (**c**), S1PR4 (**d**), and S1PR5 (**e**) in the skin of control subjects (0 h, *n* = 3; 7 h, *n* = 5; 48 h, *n* = 4; 96 h, *n* = 4) and contact dermatitis subjects (0 h, *n* = 4; 7 h, *n* = 5; 48 h, *n* = 3; 96 h, *n* = 6) (GEO: GDS 2935) were analyzed. Data are expressed as means ± SD. *, *p* < 0.05; **, *p* < 0.005; ns, not significant; S1PR1-5, Sphingosine 1-receptor1-5.

To determine if the expression of S1PR2 is altered in ACD, a genome-wide association study (GWAS) was performed. We compared gene expressions of S1PR1-5 in ACD subjects and control subjects at the time points of 0 h, 7 h, 48 h, and 96 h after allergen exposure. Based on the results of GWAS, which was conducted on human skin gene repositories at the NIH (GDS 2935) [13], S1PR2 was highly expressed upon allergen exposures after 48 and 96 h in ACD lesions compared to the baseline and control subjects. S1PR3 and S1PR4 showed increased expressions after 96 h of exposure. S1PR3 expression was not significantly different between the ACD and control groups, but there was a significant difference in S1PR4 expression. The expression levels of both S1PR1 and S1PR5 remained constant at all time points. From the data presented, it can be inferred that S1PR2 in the skin has the strongest correlation with ACD in comparison to the other four S1PRs (Figure 1).

### 2.2. Murine Skin Expresses All Five S1pr1-5 Receptor Genes (In Vivo Study)

**Figure 2:**
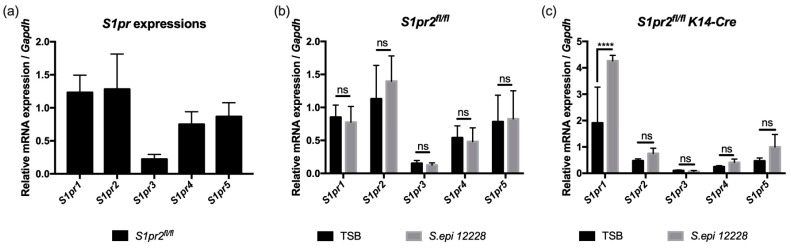
mRNA expressions of *S1pr1-5* of murine auricle skin. Total RNA was obtained from *S1pr2^fl/fl^* mouse normal ear skin (**a**), *S1pr2^fl/fl^* mouse ear skin 96 h after TSB or *S. epidermidis* application (**b**), and *S1pr2^fl/fl^ K14-Cre* mouse ear skin 96 h after TSB or *S. epidermidis* application (**c**). Expressions of five receptors for S1P (*S1pr1-5*) mRNA were measured with RT-qPCR and normalized to *Gapdh* mRNA expression level. Three mice were used per experimental group and triplicated technically during analysis. Data shown are the mean ± SD (*n* = 3) and are representative of two independent experiments with similar results. ****, *p* < 0.0001; ns, not significant; TSB, trypticase soy broth; *S. epi*, *Staphylococcus epidermidis*; *Gapdh*, glyceraldehyde-3-phosphate.

In order to confirm whether murine skin expresses all five S1P receptors, RT-qPCRs were performed using the total RNA of *S1pr2^fl/fl^* mouse ears. All five S1P receptor genes were expressed in the murine skin (Figure 2a).

In addition, to investigate whether *S. epidermidis* affects the gene expressions of S1P receptors, RT-qPCR was performed using the total RNA of *S1pr2^fl/fl^* and *S1pr2^fl/fl^ K14-Cre* mouse ears with trypticase soy broth (TSB) or *S. epidermidis* application. An increase in the mRNA expression of *S1pr1* was observed in *S1pr2^fl/fl^ K14-Cre* mice treated with *S. epidermidis*, although no changes were observed in *S1pr2^fl/fl^* mice (Figure 2b,c). This indicates that in the absence of S1PR2, *S. epidermidis* induces S1PR1 to compensate for the S1P-S1PR signaling pathway.

### 2.3. S. epidermidis Reduces Ear Edema in ACD, Especially If S1PR2 Is Absent in the Epidermis

**Figure 3:**
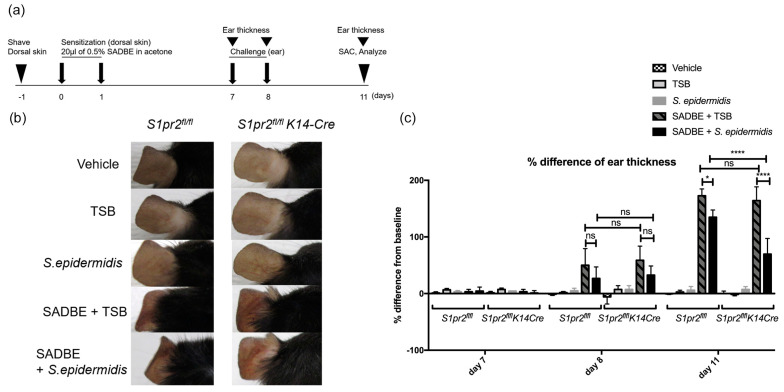
*S. epidermidis* ameliorates contact dermatitis inflammation in the *S1pr2^fl/fl^ K14*−*Cre* murine auricle skin. (**a**) Experimental protocol explaining regimen for SADBE-induced ACD. Briefly, 12-week-old mice were treated with 20 μL of 0.5% SADBE dissolved in acetone on their shaved backs for two consecutive days. Five days after, the mice ear was topically challenged with 20 μL of vehicle (acetone), or TSB or 1 × 10^6^ of *S. epidermidis,* or 0.5% SADBE/TSB, or 0.5% SADBE/1 × 10^6^ of *S. epidermidis*, respectively. (**b**) Clinical manifestations of the representatives from each experimental group on day 11. (**c**) Ear thickness was measured on days 7, 8, and 11. The difference in ear thickness after the challenge from baseline was calculated and presented as a bar graph. Three mice were used per experimental group. Data shown are the mean ± SD (*n* = 3) and are representative of two independent experiments with similar results. Data are expressed as means ± SD. *, *p* < 0.05; ****, *p* < 0.0001 ns, not significant; ACD, allergic contact dermatitis; SADBE, 3,4-Dibutoxy-3-cyclobutene-1,2-dione.

To see if *S. epidermidis* affects the inflammation of ACD, we treated 3,4-Dibutoxy-3-cyclobutene-1,2-dione (SADBE)-induced ACD mouse models and non-ACD mice with *S. epidermidis* or TSB and measured ear thickness (Figure 3a). After the challenge, ACD mice presented high inflammation with scrunched ears, while vehicle-, TSB-, and *S. epidermidis*-treated group mice without ACD displayed normal-shaped ears in both *S1pr2^fl/fl^* and *S1pr2^fl/fl^ K14-Cre* strains. However, *S. epidermidis*-treated *S1pr2^fl/fl^* ACD mice showed more folded ears compared to TSB-treated *S1pr2^fl/fl^* ACD mice. Additionally, *S. epidermidis*-treated *S1pr2^fl/fl^ K14-Cre* ACD mice had less-folded ears than TSB-treated *S1pr2^fl/fl^ K14-Cre* ACD mice (Figure 3b). Other than the folded ears, ACD mice revealed a significantly higher ear thickness than vehicle-, TSB-, and *S. epidermidis*-treated mice in both *S1pr2^fl/fl^* and *S1pr2^fl/fl^ K14-Cre* strains. However, *S. epidermidis*-treated *S1pr2^fl/fl^ K14-Cre* ACD mice showed significantly lower ear thickness than *S. epidermidis*-treated *S1pr2^fl/fl^* ACD mice (Figure 3c). These findings indicate that S1PR2 deletion in the epidermis aids *S. epidermidis* in reducing ear thickness in ACD; thus, in the absence of epidermal S1PR2 expression, *S. epidermidis* protects the skin from inflammation in ACD.

### 2.4. S. epidermidis Reduces Inflammatory Cytokine mRNA Expressions in ACD When S1PR2 Is Absent in the Epidermis

**Figure 4:**
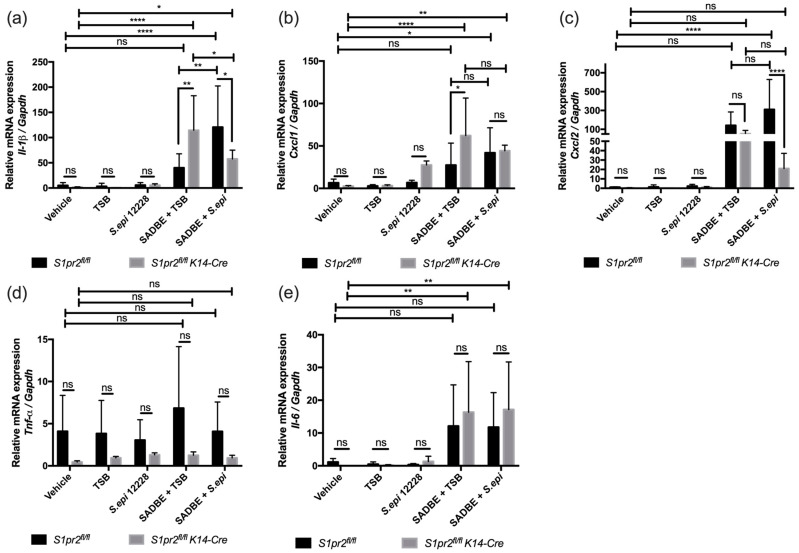
Expression of inflammatory cytokine genes in ACD mouse models and controls. mRNA expression of ACD-associated genes, *Il-1β* (**a**), *Cxcl1* (**b**), *Cxcl2* (**c**), *Tnf-α* (**d**), and *Il-6* (**e**), was measured by RT-qPCR in ACD mouse models and control mice ear skin. Three mice were used per each experimental group and triplicated technically during RT-qPCR. Data shown are the mean ± SD and are representatives of two independent experiments with similar results. *, *p* < 0.05; **, *p* < 0.005; ****, *p* < 0.0001; ns, not significant.

To determine whether *S. epidermidis* affects cytokine mRNA expression in ACD, total RNA was extracted from the ear skin of each group, followed by RT-qPCR analysis to determine mRNA expression levels of *Il-1β*, *Cxcl1*, *Cxcl2*, *Tnf-α*, and *Il-6* (Figure 4a–e, Supplementary Figure S1), which are known to be increased in ACD [14,15,16,17]. *S. epidermidis*-treated *S1pr2^fl/fl^* ACD mice had significantly higher cytokine mRNA expression levels, including *Il-1β*, *Cxcl1*, and *Cxcl2*, compared to the vehicle-treated group (Figure 4a–c), but no significant difference in *Tnf-α* and *Il-6* (Figure 4d,e). However, TSB-treated *S1pr2^fl/fl^* ACD had relatively higher cytokine expressions compared to the vehicle-treated group, but no significant difference was observed. These data indicate that *S. epidermidis* enhances rather than improves ACD in the presence of S1PR2. On the other hand, *S. epidermidis*-treated *S1pr2^fl/fl^ K14-Cre* ACD mice had increased cytokine expression, such as of *Il-1β* and *Cxcl1*, compared to the vehicle-treated group, but the difference was less significant than in TSB-treated *S1pr2^fl/fl^ K14-Cre* ACD mice (Figure 4a,b). Furthermore, *S. epidermidis*-treated *S1pr2^fl/fl^ K14-Cre* ACD mice had significantly lower *Il-1β* and *Cxcl2* mRNA expressions than *S. epidermidis*-treated *S1pr2^fl/fl^* ACD mice (Figure 4a,c). These indicate that *S. epidermidis* downregulates cytokine expression in ACD when S1PR2 is absent. Therefore, S1PR2 prevents *S. epidermidis* from suppressing skin inflammation in ACD.

### 2.5. S. epidermidis Rescues Tight Junction Expression in S1pr2^fl/fl^ K14-Cre ACD Mice

**Figure 5:**
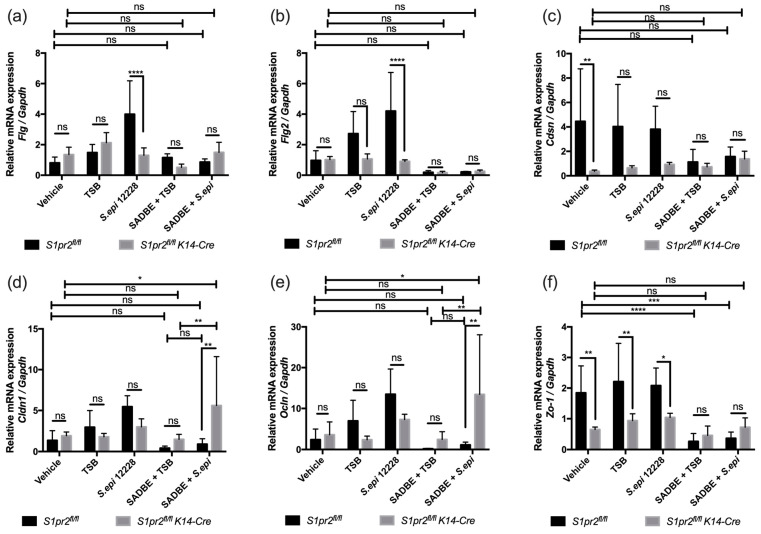
Expression of stratum corneum barrier and tight junction barrier genes in ACD mouse models and controls. mRNA expressions of *Flg* (**a**), *Flg2* (**b**), *Cdsn* (**c**), *Cldn1* (**d**), *Ocln* (**e**), and *Zo-1* (**f**) were measured with RT-qPCR in ACD mouse models and control mice ear skin. Three mice were used per each experimental group and triplicated technically during RT-qPCR. Data shown are the mean ± SD and are representative of two independent experiments with similar results. *, *p* < 0.05; **, *p* < 0.005; ***, *p* < 0.001; ****, *p* < 0.0001; ns, not significant; *Flg*, filaggrin; *Cdsn*, corneodesmosin; *Cldn1*, claudin-1; *Ocln*, occludin, *Zo-1*, zonula occludens-1.

As both S1PR2 and *S. epidermidis* have been reported to affect the skin barrier function [8,9,18], we investigated whether S1PR2 and *S. epidermidis* interaction affects the skin barrier function in ACD. According to a previous study [8], we measured mRNA expressions of *Flg*, *Flg2*, and corneodesmosin (*Cdsn*), which are components of the stratum corneum barrier, and *Cldn1*, *Ocln*, and *Zo-1*, which are components of the tight junction barrier (Figure 5a–e, Supplementary Figure S2). Similar to the previous study, *S1pr2^fl/fl^ K14-Cre* mice showed significantly lower mRNA expressions of *Cdsn* and *Zo-1* than *S1pr2^fl/fl^* mice with vehicle treatment on the intact skin (Figure 5c,f). On the other hand, no difference was observed in the mRNA expressions of all skin barrier proteins between TSB-treated *S1pr2^fl/fl^* ACD mice and TSB-treated *S1pr2^fl/fl^ K14-Cre* ACD mice. However, *S. epidermidis* increased mRNA expressions of *Cldn1* and *Ocln* in *S1pr2^fl/fl^ K14-Cre* ACD mice but not in *S. epidermidis*-treated *S1pr2^fl/fl^* ACD mice. This indicates that *S. epidermidis* increases the tight junction protein expression in ACD of S1PR2 deficient skin. (Figure 5d,e). Taken together, *S. epidermidis* ameliorates ACD inflammation in S1PR2 deficient skin through tight junction recovery.

## 3. Discussion

The cell membrane is rich in sphingomyelin and sphingolipids, and these sphingolipids play an essential role in cell membrane function. S1P is a known intermediate metabolite produced during the degradation of these sphingolipids. In the early 1990s, it was discovered that S1P has diverse effects on different types of cells, including their differentiation, proliferation, motility, and morphology, as reported previously [19,20,21]. Subsequently, five specific G-protein-coupled receptors, S1P1-5, were identified [22,23,24,25,26]. Since then, S1P receptors have been studied in relation to pathological mechanisms in various tissues and diseases. In the field of dermatology, S1P receptors have been reported to affect various skin diseases, including wound [27], scleroderma [28], epidermolysis bullosa [29], infection [30,31], and inflammatory skin diseases such as atopic dermatitis [32,33,34,35,36] and psoriasis [37,38,39,40]. Recently, Reines et al. reported that the topical application of S1P and S1P modulator, FTY720, which binds to S1PR1 and has mixed agonist-antagonist effects on S1PR1 [41], but also binds to S1PR3-5 as well, attenuated ACD reaction via inhibiting dendritic cell migration [42]. However, the influence of S1PR2 in the epidermis and ACD remained to be elucidated. This study showed that deleting epidermal S1pr2 increased the expression of *Il-1β* and *Cxcl1* in ACD. However, there was no significant increase in ear swelling in comparison to control mice. This suggests that epidermal S1pr2 deletion leads to increased inflammation in the epidermis but does not affect dermal edema.

A compromised skin barrier can increase the risk of developing ACD. This is because a damaged skin barrier allows allergens to penetrate more quickly into the skin, where they can trigger an immune response [43]. According to published studies, tight junction and stratum corneum protein gene expressions, including CLDN-1, OCLN, FLG-1, and FLG-2 in the skin of patients with severe ACD, are downregulated [44]. Additionally, patients deficient in the FLG gene develop contact dermatitis at lower allergen exposure than patients carrying the FLG wild-type gene [45]. Furthermore, tight junction repair helps alleviate ACD inflammation [46]. Therefore, to reduce the risk of ACD, various strategies for the use of emollient creams and moisturizers have been developed to protect the skin barrier function [47].

The skin barrier is mainly composed of stratum corneum and junctions of nucleated epidermis [48]. FLG is the most expressed protein to form the stratum corneum [49], and CDSN is a protein that forms corneodesmosome, which adheres corneocytes to each other [50]. The tight junction is one of the junctions of the nucleated epidermis, composed of OCLN, junctional adhesion molecules, CLDNs, and cytoplasmic scaffolding protein ZO [51,52]. Our previous study found that S1PR2 knockout mice have a decreased mRNA expression and production of skin barrier proteins, including FLG, CDSN, CLDN1, OCLN, and ZO-1 in shaved back skin. However, it remains to be examined whether S1PR2 in the epidermis or S1PR2 in other parts affect skin barrier maintenance [8]. In this study, we investigated the relationship between skin barrier function and S1PR2 in the epidermis, using murine auricle skin. Our results suggest that S1PR2 deletion decreased *Cdsn* and *Zo-1* expression in the intact skin. However, S1PR2 deletion caused no significant difference in the skin barrier expressions in ACD mice (Figure 5, SABDE + TSB). This study suggests that the presence of S1PR2 in the epidermis is crucial for forming the skin barrier, although it does not significantly impact ACD.

Recently, a large number of studies have been conducted to study the relationship between the skin microbiome, skin commensal bacteria, to be more specific, and skin disorders. *S. epidermidis,* a type of human commensal bacteria, is shown to either improve or disrupt the skin barrier, depending on the skin condition [53]. Based on the published study, *S. epidermidis* contributes to skin barrier maintenance by secreting sphingomyelinases, which have an essential role in generating ceramides [9]. In addition, a randomized controlled trial showed that microbiome-supporting skincare contributes to increasing *S. epidermidis* and improves biophysical skin parameters [54]. This study also showed that *S. epidermidis* increases the mRNA expressions of stratum corneum skin barrier proteins in the steady state, supporting these reports (Figure 5, *S. epi* 12228). Some strains of *S. epidermidis* can downregulate skin barrier molecules such as FLG and induce proinflammatory cytokines associated with atopic dermatitis [18]. In our model, we did not notice significant changes in ACD skin (Figure 5a–d, SADBE + *S. epi* black column and SADBE + TSB). Moreover, an overabundance of *S. epidermidis* on atopic skin disrupts the skin barrier and induces skin damage and inflammation [55]. In addition, the excess colonization of *S. epidermidis*, which promotes protease production in the epidermis, is formed in Netherton’s syndrome, where the skin barrier function is impaired [56]. Recently, it has been reported that the effect on the skin barrier from *S. epidermidis* depends on the skin type; the *S. epidermidis* strain from healthy skin maintains the epidermal barrier, while the strain from atopic dermatitis decreases the epidermal barrier, using ATCC12228 and *S. epidermidis* strains isolated from hyper-sebaceous or atopic skin [57]. According to this study, *S. epidermidis* can help restore the tight junction barrier in ACD skin when S1PR2 is lacking. This implies that when S1PR2 is absent, *S. epidermidis* can aid in repairing the impaired skin barrier.

A potential explanation for the results of the study is that *S. epidermis* produces S1P through its sphingomyelinase activity [9] and that environmental stress stimuli such as allergen exposure activate sphingomyelinase [58]. Our study found that in the absence of S1PR2, *S. epidermidis* induces S1PR1 increase in the skin (Figure 2b,c). In the absence of S1PR2, elevated S1PR1, induced by *S. epidermidis*, compensates for the lack of S1PR2 signal and protects from allergen exposure that occurs in ACD. Recently, Yanagida et al. reported that cytoskeletal claudin and occludin decreased in the absence of S1PR1 [59] without S1PR2, so S1P may prioritize targeting S1PR1, which has a defensive impact against ACD, as reported previously [42].

In order to investigate the possible cross-talk mechanism between S1PR2 and *S. epidermidis,* further research is highly demanded. Additionally, it is necessary to perform experiments using different strains of *S. epidermidis*, including *S. epidermidis 1457*, *S. epidermidis* from healthy skin, and *S. epidermidis* from ACD skin, to confirm if all strains affect the skin barrier or only certain ones.

To summarize, our research suggests that *S. epidermidis* plays a beneficial role in restoring the skin barrier of damaged skin. This is achieved by restoring tight junctions when S1PR2 cannot compensate for the damage, as observed in human ACD and depicted in Figure 1. When S1PR2 is lost, the signaling of S1P-S1PR becomes opposed by other S1PRs, with S1PR1 being the most significant. For this reason, focusing on the interaction between S1PR2 and S. epidermidis may result in a remedy for ACD.

## 4. Materials and Methods

### 4.1. Reagents

Acetone and SADBE were obtained from Sigma-Aldrich (St. Louis, MO, USA).

### 4.2. Bacterial Culture

*S. epidermidis* (ATCC Catalog No. 12228) was acquired from ATCC. Bacterial stocks were frozen at −80 °C in trypticase soy broth (TSB, Catalog No. U363, Hardy Diagnostics, Santa Maria, CA, USA) with 20% glycerol (Catalog No. G7893, Sigma-Aldrich, St. Louis, MO, USA). To grow bacteria, 1 μL of bacterial stock was cultured in 2 mL of TSB for 15–16 h at 37 °C while shaking at 300 rpm with the lid of the culture tube loosely closed. For scale-up preparation, cultures were diluted in 1:100 with TSB and were cultured for 15–16 h more at 37 °C while shaking at 300 rpm without closing the lid completely. The cultured bacteria were measured as OD600 with a spectrophotometer UNICO 1100 (UNICO, Dayton, NJ, USA). OD600 = 1 was equal to 3 × 10^8^ CFU/mL.

### 4.3. Mice

*S1pr2^fl/fl^* mice were gifted from Dr. Hla’s laboratory at Boston Children’s Hospital (MA, USA). *K14-Cre*-transgenic mice were gifted from Dr. Gallo’s laboratory at the University of California San Diego (CA, USA). Epidermis-specific S1PR2 knockout mice (*S1pr2^fl/fl^ K14-Cre*) were generated by crossing *S1pr2^fl/fl^* mice with *K14-Cre*-transgenic mice of C57BL/6 background. Approximately 50% of the offspring were heterozygous for the *loxP* allele and heterozygous for the Cre transgene. They were then backcrossed with homozygous *S1pr2^fl/fl^*. Approximately 25% of the offspring were homozygous for the *loxP* allele of *S1pr2* and heterozygous for the *Cre* transgene.

All mice were housed in the laboratory animal facility at the University of California San Diego in individual ventilated plastic cages and were provided food and water ad libitum under temperature-controlled conditions with a 12 h light/dark cycles. All animal protocols were reviewed and approved by University of California San Diego (IACUC approval number: s10288).

### 4.4. Development of Contact Dermatitis Mouse Models

Twelve-week-old mice were divided into five groups (*n* = 3): an acetone-treated control group as a vehicle control group, a TSB-treated group, the *S. epidermidis*-treated group, the SADBE/TSB-treated group, and the SADBE/*S. epidermidis*-treated group. To induce contact dermatitis, 1 cm^2^ of gluteal skin had hair removal performed via shaving and hair removal cream application on the day before sensitization. On days 0 and 1, 20 μL of 0.5% SADBE was dissolved in acetone and applied to the shaved gluteal skin once daily. On days 7 and 8, mice were challenged with the application of 20 μL of 0.5% SADBE in acetone to the left ear once a day. The vehicle-, TSB-, and *S. epidermidis*-treated groups were treated topically with 20 μL of acetone, or TSB, or 1 × 10^6^ CFU/20 μL of *S. epidermidis* in TSB instead of SADBE on days 7 and 8. The SADBE/TSB-treated group was topically treated with 20 μL of TSB after SADBE application on days 7 and 8. The SADBE/*S. epidermidis*-treated group was topically treated with 1 × 10^6^ CFU/20 μL of *S. epidermidis* in TSB. The ear thickness was measured on days 7, 8, and 11 using Kynup Digital Caliper (Deqing Liang Feng Electronic & Technology Co., Ltd., Zhejiang, China). The ear skin was collected for further analyses on day 11.

### 4.5. Real-Time RT-qPCR

Total mouse RNAs from mouse ear skin samples were isolated using a Quick RNA Miniprep Kit (Catalog No. R1055, Zymo Research, Irvine, CA, USA). At least 1 μg of total RNA was converted to cDNA with a High-Capacity cDNA Reverse Transcription Kit (Catalog No. 4368814, Applied Biosystems, Waltham, MA, USA), according to the manufacturer’s instructions, on a T100™ Thermal Cycler (Catalog No. 1861096, Bio-Rad Laboratories, Hercules, CA, USA). Real-time RT-qPCR was performed using TaqMan™ Fast Advanced Master Mix (Catalog No. 4444557, Applied Biosystems, Waltham, MA, USA) or PowerUp™ SYBR™ Green Master Mix (Catalog No. A25742, Applied Biosystems, Waltham, MA, USA) on Quant Studio™ 3 Real-Time PCR Systems (Catalog No. A28132, Applied Biosystems, Waltham, MA, USA). The primers and the probes used for real-time RT-qPCR are listed in Supplementary Tables S1 and S2, respectively. The expression of target genes was normalized to *Gapdh* expression and analyzed with the 2^−ΔΔct^ method [60].

### 4.6. Genome-Wide Association Studies

In all experiments, all groups had at least three subjects, and values were expressed as mean ± SD. The statistical significances of differences were determined with two-way ANOVA and Tukey’s multiple comparison test. *p* < 0.05 was considered significant (*, *p* < 0.05; **, *p* < 0.005; ***, *p* < 0.001; ****, *p* < 0.0001). Analyses were performed through the GraphPad Prism software version 7 (GraphPad Software, Boston, MA, USA).

### 4.7. Statistical Analysis

In all experiments, all samples were performed in triplicate, and values were expressed as mean ± SD. Statistical significances of differences were determined by two-way ANOVA and Tukey’s multiple comparison test. *p* < 0.05 was considered significant. Analyses were performed through the GraphPad Prism software version 7 (GraphPad Software, Boston, MA, USA).

## Data Availability

No novel datasets were generated in this study. Patient biopsy datasets are available at Gene Expression Omnibus (GEO) repository (http://ncbi.nlm.nih.gov/gds), published on 3 April 2007, with accession code GDS 2935.

## Supplementary Materials

The following supporting information can be downloaded at: https://www.mdpi.com/article/10.3390/ijms241713190/s1.
