# Supplementary figures and images for: S. epidermidis Rescues Allergic Contact Dermatitis in Sphingosine 1-Phosphate Receptor 2-Deficient Skin
Source: Int J Mol Sci. 2023 Aug 25;24(17):13190. doi: 10.3390/ijms241713190 (PMC10487941; doi:10.3390/ijms241713190)

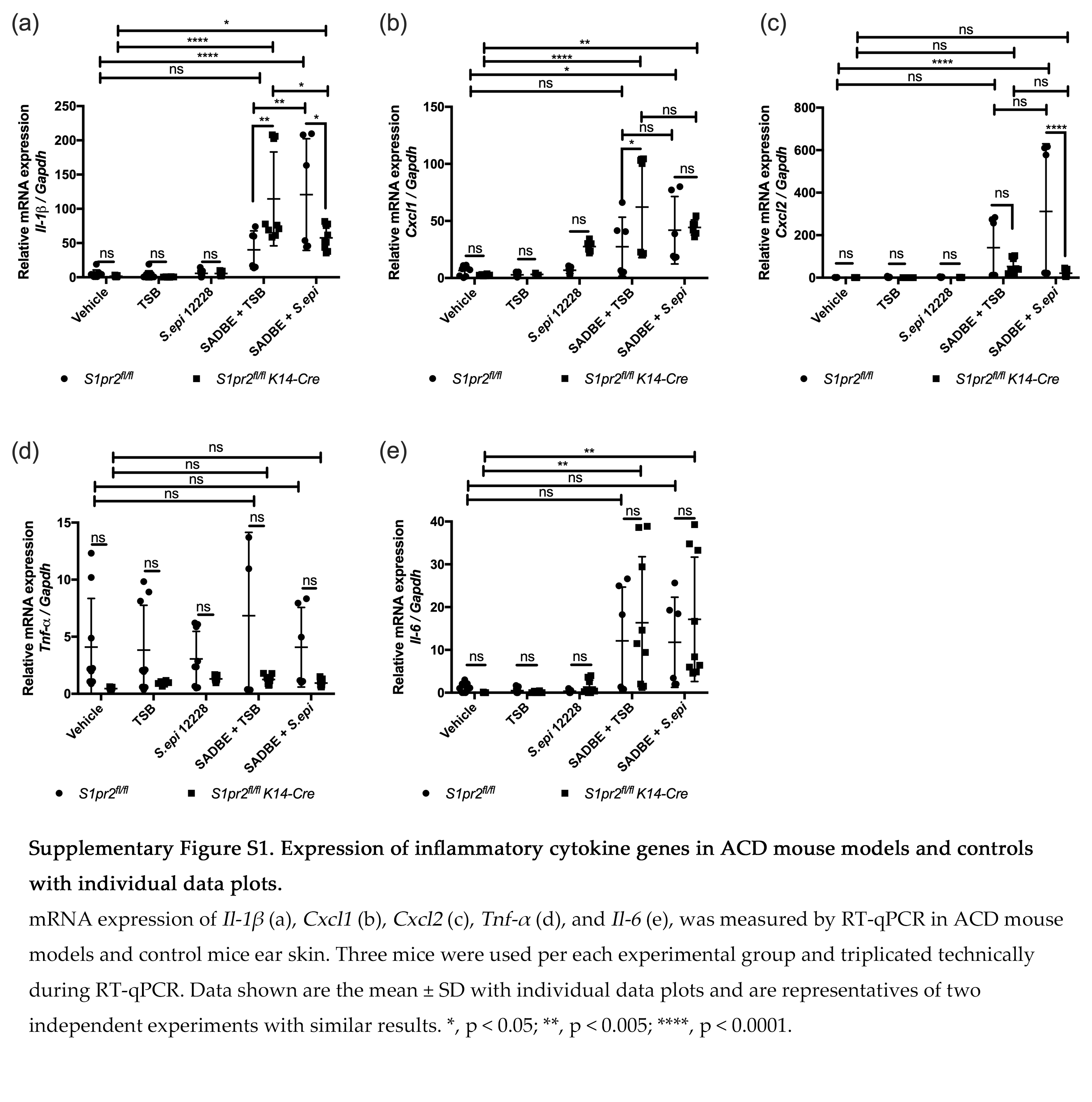

Supplement: Supplementary file 1 [file ijms-24-13190-s001.zip › Supplementary Figure S1.tif]

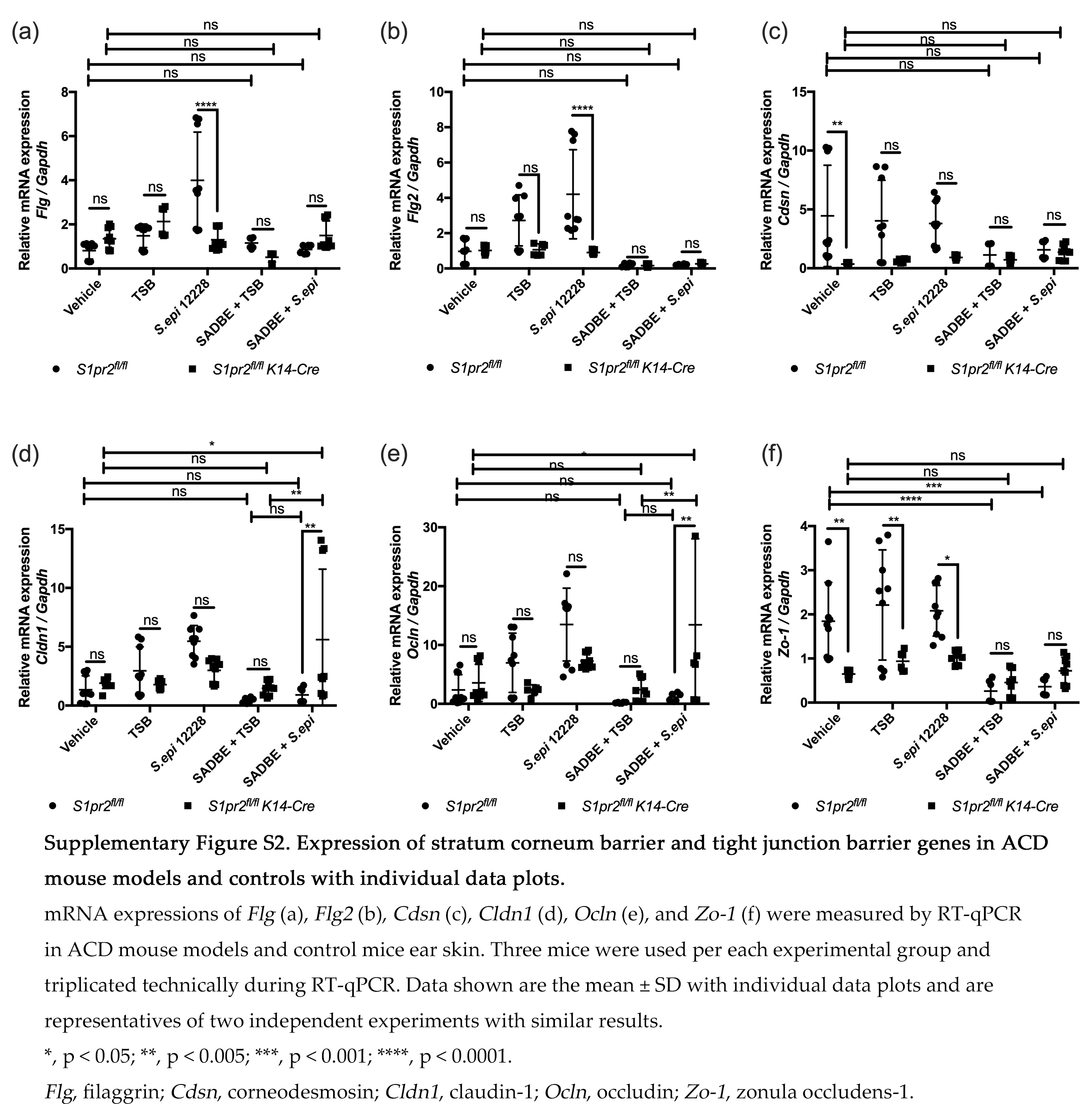

Supplement: Supplementary file 1 [file ijms-24-13190-s001.zip › Supplementary Figure S2.tif]
